# Prospects for AI clinical summarization to reduce the burden of patient chart review

**DOI:** 10.3389/fdgth.2024.1475092

**Published:** 2024-11-07

**Authors:** Chanseo Lee, Kimon A. Vogt, Sonu Kumar

**Affiliations:** ^1^ Department of Surgery, Yale School of Medicine, New Haven, CT, United States; ^2^ Sporo Health, Boston, MA, United States

**Keywords:** artificial intelligence, patient chart review, clinical summarization, LLM, electronic health records, cybersecurity, data privacy

## Abstract

Effective summarization of unstructured patient data in electronic health records (EHRs) is crucial for accurate diagnosis and efficient patient care, yet clinicians often struggle with information overload and time constraints. This review dives into recent literature and case studies on both the significant impacts and outstanding issues of patient chart review on communications, diagnostics, and management. It also discusses recent efforts to integrate artificial intelligence (AI) into clinical summarization tasks, and its transformative impact on the clinician’s potential, including but not limited to reductions of administrative burden and improved patient-centered care. Furthermore, it takes into account the numerous ethical challenges associated with integrating AI into clinical workflow, including biases, data privacy, and cybersecurity.

## 1 Introduction

Patient information is critical in the delivery of effective care – thousands of practices, tools, and techniques have been developed in patient interview, health record storage, and physical examination purely for the sake of effective usage of key patient information. Clinicians must have an effective understanding of a patient – including but not limited to the history of present illness (HPI), past medical history (PMH), family history (FH), and more. This allows them to discern accurate differentials and develop efficacious management plans.

In modern healthcare, collected patient information is stored in electronic health records (EHRs), where they lie unstructured across thousands of progress notes, lab results, office visits, phone call transcriptions, and the like. Patient chart review, also known as pre-charting, involves condensing this unstructured information into an accurate picture of a patient’s medical history and current health status into a concise, accessible format. This practice has significant implications for healthcare professionals, patient health outcomes, and hospital expenditures. However, even in a strictly regulated industry that is American healthcare, clinicians have diverse ways of approaching patient chart review – with many placing much time, energy, and value, while others not so much.

The process of condensing medical information from various sources, including but not limited to biomedical texts, literature, and patient information, is known as clinical summarization. Patient chart review is a subset of clinical summarization, but arguably one of the most important in the field. Tools that streamline clinical summarization have been a hot topic of debate, with many arguing for its effectiveness in healthcare delivery while others fear issues in data privacy, ethical considerations, and more. This debate is further complicated with the advent of generative AI and its impact on workflows across the industry. However, it is no surprise that AI that automates clinical workflow is an exciting frontier. AI-powered clinical summarization is finding its foothold cautiously in the hands of physicians and slowly into clinical practice. This review article will explore the current state of patient chart reviewing in healthcare, and how AI pushes its frontiers to previously unexplored heights.

## 2 Quantifying patient chart review’s impact on diagnostic accuracy and time burden

Patient chart review is clinical summarization of unstructured patient health data lying in EHRs through interpretation of salient information. It is an essential part of any clinical workflow, regardless of clinician, specialty, or patient. Reviewing EHRs allows for the physician to focus on talking with patients effectively by gaining contextual information about the patient (1). The wealth of information housed within the patient charts is just as critical as the patient interview, physical examination, or lab/imaging workups, especially to avoid misdiagnoses or contraindicatory management plans.

In fact, this valuable nature of EHR data is precisely why there are many efforts to implement natural language processing of clinical narratives into both workflow and diagnostics, including in managing coronary artery disease, depression, and more (2, 3). Especially for patients with chronic conditions, it is generally agreed that clinical free-text, or the unstructured narrative information lying inside the health records, is dominant in value over any of the other structured data such as ICD-10 codes, which are often plagued with errors/misdiagnoses (4). Thus, it should only be natural that literature on EHR have discovered that low quality medical data management and usage are key reasons for medical error (5).

One case study has also shown that unstructured, clinical narrative information contained in EHRs for patient chart review is sufficient for conclusions about the patients’ pathophysiological processes and therapeutic advances, even for up to 94.9% of cases. In fact, thorough patient chart reviews can take up to 30 min per patient case, but this time investment can have high returns, identifying most or even all of the major patient issues correctly in up to 93.8% of the cases (6).

However, even while taking 30 min per patient to conduct a thorough chart review, the diagnostic and management decisions are not perfect – an independent, second round of patient chart review evaluating the accuracy of the first round of patient chart review found that 36.6% of the cases had to be corrected in either the pathophysiological process identification or therapy/management decisions, highlighting the imperfection of even the most thorough patient chart review process (6).

## 3 Medical errors associated with patient chart review

Most physicians in the United States do not take sufficient time to conduct patient chart review. A survey of 155,000 U.S. physicians in ambulatory subspecialties or primary care utilizes 5 min and 22 s per patient encounter (7) – a significant time sink, but nowhere near the 30 min average used in the aforementioned case study. The poorer quality of the average patient chart review, whether it be due to work burden overload, lack of time, or negligence, leads to larger quantities of misdiagnoses, and thus, medical errors/malpractice.

There are many medical error scenarios associated with patient chart review. For example, a common case of medical error are iatrogenic adverse drug events (ADE), most commonly caused by inconsistencies in a clinician’s knowledge on patient allergies to medications. There is a wealth of literature and case studies that review these adverse drug events caused by insufficient documentation, poor patient health record communication, and lack of proper information collection from charts. As these case studies show, many of these ADEs involve insufficient knowledge on the side of a clinician due to incomplete record review and internal inconsistencies found in the unstructured data within health records (8). In fact, another study highlights this lack of clinician knowledge despite sufficiently documented patient information, having caused 29% of the study’s preventable ADEs (9).

Another sector where patient chart review is key is transition of care. Patients being transferred between clinicians, whether internally in hospital systems or across practices, require fluid and comprehensive communication of all relevant patient health history to prevent confusion, poor management, and ultimately malpractice or negligence. An average large academic teaching hospital can have up to 4,000 transitions per day, and this high volume of transition is a rich breeding ground for medical errors due to lack of comprehensive patient information and thus, a poor understanding of a patient’s status (10). In fact, a 2016 study showed that 30% of malpractice claims in the U.S. were attributed to poor communication between clinicians, resulting in 1,744 deaths and $1.7B in claims (11).

## 4 Information overload and physician burnout

The root driver behind patient chart review causing medical errors has been investigated quantitatively through literature. On the other side of the patient-physician interaction, consider the burden of information placed on the physician. Each patient can have patient records as short as 29 pages and as long as over 500 pages long (12), and it is also important to note that with note redundnacy and length on the rise since this 1995 study, this number is a severe underestimate. To paint an accurate picture from this daunting data is a monumental manual task. A physician spends an average of 4.5 h per day doing EHR workflow, with 33% of that being patient chart review, translating to 1.5 h of patient chart review per day (7). Even the average U.S. medical resident spends 112 h per month exclusively reading patient charts (13).

**Figure 1:**
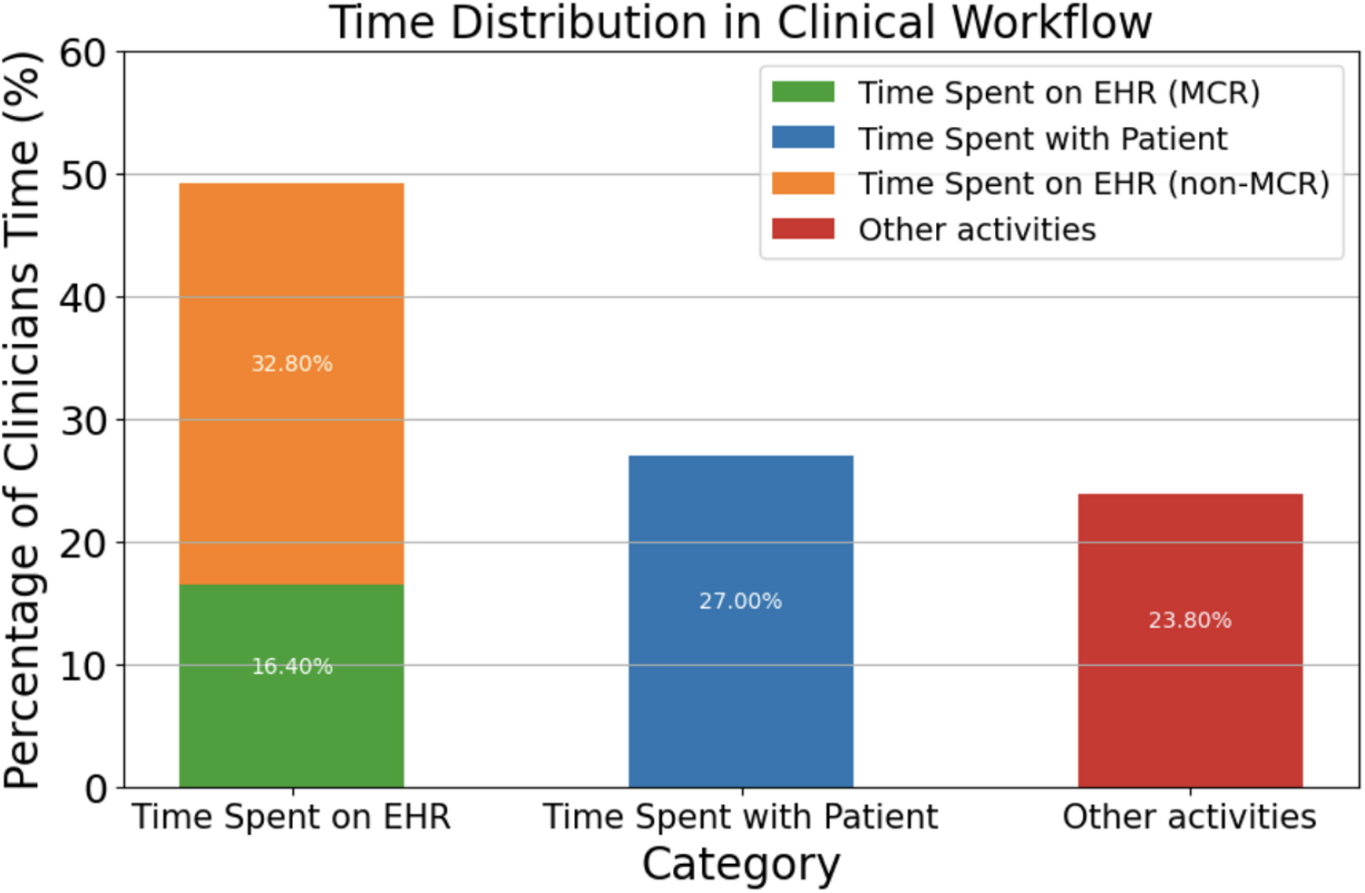
Time distribution in clinical workflow.

Electronic health records directly contribute to what has been dubbed as “information overload crisis,” where physicians actively face an excess of information from patients, research, and administration (14). In fact, studies have shown that 75% of physicians facing burnout cite EHR workflow as the main contributor (15), especially in primary care, where burnout rates are the highest at 50%. This high correlation between burnout and EHR workflow can be attributed to the fact that physicians spend 49.2% of their time per day with EHRs while only 27% is dedicated towards face-to-face time with patients (16) (Figure 1).

It is difficult to quantify exactly what portion of medical errors are caused by problems with the information crisis and electronic health records. However, it is still possible to discern what the errors that were made from information handling processes, which heavily involve patient charts. In fact, one family medicine case study found that 29% of the errors made can be associated with patient information processing. These errors include the availability of information within patient charts, physician-physician communications, and clinical knowledge gaps (17).

Another study of 2,590 primary care physicians showed that 69.6% receive more information that they can handle. This study measured the number of alerts a physician received, which is a common proxy for measuring information overload. Furthermore, these alerts lead to almost 30% of these physicians reporting missing test results and delayed patient care as a result, another proxy for medical errors due to the information overload (18). These studies highlight the burden of information placed on the physician, and how it impacts not just their time usage, but also prevalence of medical errors and physician burnout.

Current solutions to combat information overload has been mainly reliant on human agents and intermediaries - to dedicate more manpower and networking to distribute the burden of information over multiple individuals. Partitioning these tasks to convert unstructured data from patient health information, biomedical literature, and the like, to structured information has primarily been only been done by trained healthcare professionals and trainees. Certain software solutions in the past have been proposed, but ultimately, the variance associated with healthcare delivery across systems, clinicians, and even individual patient cases, proved to be a daunting challenge to be solved by traditional software, and has mainly fell on the clinical team members to handle (19).

## 5 The role of AI in healthcare support and clinical summarization

The growing crisis in healthcare information volume, physician burnout, and patient-physician relations, increasing efforts to incorporate artificial intelligence into patient chart review. Natural language processing (NLP) can be used to determine illnesses or patient information from clinical free-text (20). The increasing capabilities and token storage of LLMs in 2024 such as Google’s Med-Gemini, Meta’s Llama 3, OpenAI’s ChatGPT4, or Anthropic’s Claude 3.5, has allowed for these models to process the enormous portions of information for summarization and analysis. In recognition of the stringent accuracy, the need for personalization, privacy regulations, and the high knowledge floor needed for AI in clinical workflow, the innovation space gave birth to many ventures to combat the aforementioned issues in clinical summarization. Several case studies verify AI usage in various clinical settings to aid in chart reviewing. The AI models and data sources investigated in each of the following case studies are summarized in Table 1.

**Table 1:** Summary table of case studies in AI applications.

Study	Models tested	Additional modifiers	Data source	Evaluation metric
1	GPT-3davinci, BARTcnn, T5, LED	None	CNN+DailyMail, booksum, sec-litigation, MIMIC-III	ROUGE, BERTscore, text length reduction
2	GPT-4, GPT-3, FLAN-T5, FLAN-UL2, Llama-2, Vicuna	ICL, QLoRA	Open-i	ROUGE, BERTscore, clinician evaluation
3	FLAN-T5, BERT, pegasus-xsum	SPeC	MIMIC-CXR	ROUGE, BERTscore
4	Med-BERT	Prediction head, fine-tuning via smaller data sets	Cerner, Truven	Disease Prediction AUC

### 5.1 Case study 1: radiology case reports

Radiological reports, essential for diagnosing and monitoring diseases, can be lengthy and complex, often integrated into almost every progress note. While the data is more structured than typical progress notes, there is much to unpack in what is necessary information and what is not.

Chien et al. utilized NLP summarization models from various sources for the purpose of summarizing neuroradiology case reports and charts. These included models such as BARTcnn, trained on news datasets from CNN, LEDClinical, trained on references from the MIMIC-III dataset, and even GPT3 davinci from OpenAI (21). Both clinical-sided physician evaluation of comprehensibility, accuracy, redundancy, and readability as well as standard AI-sided quantitative evaluation using ROUGE or BERTscore (22, 23) was performed on the summarization capabilities of these models.

These AI models, especially BARTcnn and GPT3 davinci, demonstrated considerable summarization capabilities, enhancing the readability and comprehensiveness of summaries, while simultaneously reducing text length to less than 20% of the original case reports. These results are especially notable when considering that most of the models tested were not trained on any clinical dataset, which opens much potential for using these AIs as tools for enhancing clinical workflow in fast-paced clinical settings (21).

However, the performance of these models did not come without limitations. For one, a common challenge associated with LLM-based patient chart reviewing is that it is difficult to guarantee the comprehensiveness of summaries, and the models in this study often left out critical information at the end of reports, typically patient outcomes or treatments. Furthermore, different models tend to leave out different pieces of information for unknown reasons, such as aneurysm locations. However, considering that most of the models were not clinically oriented, nor trained on clinical data, this case study still demonstrates great promise for the capabilities of NLP models to greatly improve clinical workflow efficiency (21).

### 5.2 Case study 2: capabilities of large language models with patient charts

Van Veen et al. recently published in Nature Medicine a study that evaluates leading Large Language Models (LLMs) in their ability to summarize clinical information in patient charts (24). Eight open source and proprietary models including ChatGPT3.5, ChatGPT4, LLaMa-2, Med-Alpaca, which were then adapted to the summarization tasks at hand using in-context learning (ICL) and quantized low-rank adaptation (QLoRA), were evaluated in its capabilities to summarize progress notes, radiology reports, dialogue, and other patient-sided sources of information. Datasets utilized for patient chart review tasks included MIMIC-III, MIMIC-CXR, and ProbSum. The study found that the best-performing models, namely ChatGPT4 adapted with ICL, performed superior to even physicians when evaluated both on the AI-sided metrics and clinical-sided expert evaluations by other physicians. In fact, a common challenge when discussing LLM outputs are hallucinations, or outputs that may be coherent yet contain nonsensical or factually incorrect information, as if the model decided to ”make up” information to compensate for the lack of a proper answer. However, data in this study suggests that adapted LLMs may even outperform humans in avoiding hallucinations, thus reducing mistakes in clinical summarization of patient charts.

However, this study, and LLMs in general, do not come without limitations. Another key challenge of implementing AI into clinical workflow is the diversity of practice that varies between not just specialties, but individual clinicians. Different information can be stressed across specialties, such as cardiology focusing on cardiac symptoms, or neurology focusing on neurological symptoms. However, this problem could be overcome through further training children models with more data, or fine-tuning to fit each specialty (24). Another possibility for fine-tuning summaries is train models to fit to each clinician’s style through real-time clinician inputs and feedback.

A second, more pressing reality with incorporating LLMs is handling context and proper referencing. Notes in electronic health records face two challenges: note length and note redundancy. A cross-sectional study by Rule et. al of almost three million outpatient progress notes revealed that the median length of notes is 642 words in 2018, increasing by 60.1% since 2009 (25). The most commonly used model in 2024, OpenAI’s GPT-4o, has a context window of 128,000 tokens, or about 96,000 words (26). This means that a patient with more than a mere 149 notes already exceeds the context window allocated by GPT-4o. Even allowing the possibility of older, obsolete notes, the problem of processing which notes are of relevance *a priori* is a distinction without a difference to the original task.

Furthermore, Rule et al. also discovered that note redundancy reached over 50% in 2018, further exacerbating the problem of overrunning context size (25). Similarly, this problem cannot be overcome without processing each note individually for redundancy, a daunting manual or programming task. This is especially true considering that information may be redundant but the actual associated files may have many differences, even with text portions that were transferred verbatim. These problems highlight a need for both innovation and a paradigm shift in electronic health records.

While there are significant challenges associated with utilizing leading models in clinical workflow, there still lies significant potential of large language models, such as ChatGPT4 adapted with in-context learning, to enhance clinical summarization tasks. These models have shown an impressive ability to process and summarize complex medical data with accuracy that sometimes surpasses human performance, particularly in reducing hallucinations. That being said, challenges such as handling diverse clinical practices and managing the vast and often redundant information in electronic health records highlight the need for further refinement. There is also a need to address context window limitations because of the modern problem of note length and note redundancy, and they remain critical hurdles in the effective integration of AI into clinical workflows. As these models continue to evolve, their ability to tailor outputs for individual clinicians and specialties could further solidify their role in healthcare, provided these limitations are addressed through continued research and innovation, and perhaps even a change in the way healthcare approaches electronic health records.

### 5.3 Case study 3: addressing variability in AI outputs for clinical summarization tasks

Beyond the typical usages of AI in clinical summarization, there have also been efforts to improve AI performance in clinical settings. Chuang et. al recently presented the SPeC framework, which represents a breakthrough in addressing the variability of AI outputs in clinical summarization of radiology reports. By employing soft prompts, this method enhances the stability and consistency of AI-generated summaries, which is critical for clinical accuracy and reliability. The framework aims to mitigate the impact of prompt quality on the performance of LLMs, demonstrating a novel approach to improving AI utility in healthcare (27).

However, this study highlights several key limitations in using large language models (LLMs) like SPeC for clinical notes summarization. One of the main challenges is the performance variability across different clinical domains and note types, especially when it comes to more specialized knowledge. While LLMs demonstrate strong performance on certain types of clinical documentation, such as structured reports, they often struggle with unstructured or highly specialized notes, which require a deeper understanding and interpretation. In fact, even nuances such as medical abbrieviations and complex medical terminologies could mislead the models utilized in the SPeC study. Similarly to Van Veen et al., the study also points to limitations in soft prompt-based calibration when attempting to standardize LLM outputs across diverse clinical tasks, as prompts may not sufficiently capture the context or detail needed for accurate summarization in all scenarios (24, 27). Moreover, ethical and practical concerns around the use of AI in healthcare, such as the potential for hallucinations, bias, or incorrect information, remain significant barriers to widespread adoption, despite advances in model training. This will be discussed in more detail later in this review.

### 5.4 Case study 4: data, frameworks, and fine-tuning

One key example of clinical datasets is the aforementined MIMIC series, including MIMIC-III, MIMIC-IV and MIMIC-CXR (28), which contains extensive patient reports, clinical information, radiological reports, and images. Many models, including G-BERT, a combination of the BERT framework and Graph Neural Networks, are trained off of the MIMIC-III dataset (29).

Rasmy et al. takes the current work on utilizing BERT and takes it a step further. The study used the Cerner Health Facts and Truven Health MarketScan datasets to create Med-BERT. With additional fine-tuning datasets, Rasmy et al. achieved higher predictive accuracy on disease prediction, increasing AUCs by up to 20 percent compared to using gated recurrent unit networks alone. They were even able to achieve accuracies on par with models that were trained on datasets ten times larger. However, this study, as with many others, come with significant limitations. The most salient for discussion is that the models were trained off of structured datasets, which led to Med-BERT being trained on pre-structured diagnostic information (in ICD format) instead of off of unstructured information in clinical notes. The information overload crisis that clinicians face everyday is often in the challenge of converting unstructured information into a structured summary. As such, we see that while studies like these present the power of patient chart review and diagnostics, it still fails to overcome the key challenge of structuralizing data, where a bulk of the time and energy burden lies (29).

There are many other clinical text or imaging databases for clinicians and scientists to train AI/ML models. Thousands of clinical notes are made available by the i2b2, which released a series of NLP challenges and the respective data for each challenge, which comprises of deidentified discharge summaries (30). Furthermore, clinical trials data is available in various digital datasets, including the PRO-ACT database, which provides access to Phase II and Phase III clinical trials data from ALS patients (31). Finally, imaging data can be extracted from a multitude of nationally funded institutes, including but not limited to the NIH National Cancer Institute’s TCIA (32) and OASIS, which feature open access to the data used in its MRI studies (33).

The challenge of data availability also extends across international scales. Most models in clinical workflow and diagnostics are trained on data produced in the United States and China, which produces challenges in output accuracy for patients who fall outside those demographics. Key differences in population-level health across international borders makes it crucial for proper data production, labeling, and pre-processing before training any model. For example, utilizing data from gastrointestinal (GI) patients in the United States may not be the most representative for utilizing with Korean patients, who experience a much higher rate of GI diseases than the rest of the world. While the continued data produced by these countries is paramount to furthering our understanding of AI development, it is crucial to also emphasize data collection from diverse sources that may lack representation (34).

While all the datasets mentioned in this review have significantly advanced AI-driven clinical summarization and disease prediction, they still face substantial challenges related to data structure and representativeness. Models like Med-BERT, which rely heavily on structured clinical data, demonstrate the power of current datasets but highlight the limitations in converting unstructured clinical notes into usable formats for training AI. Moreover, the reliance on datasets predominantly from the United States and China underscores the need for more diverse, global data collection to enhance the applicability and accuracy of AI models across different populations. Addressing these challenges requires concerted efforts in data preprocessing, international collaboration, and the development of methods to structure unstructured clinical data, which remains a critical bottleneck in AI-driven healthcare innovation.

## 6 Ethical challenges in AI-driven clinical support

Incorporating AI into clinical summarization and patient chart review presents significant ethical challenges, particularly concerning bias, patient privacy, and cybersecurity. Algorithmic bias, which arises when AI systems are trained on non-representative or imbalanced data, potentially leading to unequal healthcare outcomes for marginalized groups. Another critical issue is patient privacy and informed consent. The use of AI often requires large datasets, raising concerns about how patient data is stored, shared, and protected. Further issues include cybersecurity risks of cloud-based architectures and adversarial attacks on LLMs for reverse data extraction. While anonymization techniques are commonly used, the risk of re-identification and unauthorized access persists, which could compromise patient confidentiality. Addressing these ethical concerns is crucial for ensuring that AI enhances, rather than hinders, clinical practice.

### 6.1 Risks of biases in utilizing AI for clinical support

Bias in AI-driven clinical summarization is a pressing issue that can exacerbate existing healthcare disparities. One of the main sources of bias comes from the datasets used to train AI models. Many of these datasets are not representative of the broader population, often over-representing certain demographic groups while under-representing others. For example, models for breast cancer are trained on more data with women, and therefore produce clinically and ethically significant differences in decision-making between male and female-born patients (35). These statistical machines with limited data diversity can produce to clinical summaries that do not accurately reflect the conditions or treatments relevant to underrepresented groups, potentially skewing decision-making in healthcare contexts.

Additionally, AI algorithms often reflect the biases present in clinical practices, such as diagnostic disparities between men and women or between racial groups. For example, women may be treated differently for cardiovascular conditions than men due to various factors, including differential auscultation practices, which can introduce significantly biases in data (36).

It is also important to mention that biases don’t necessarily have to exist just from data, but purely from the availability of technology, either in development or distribution. For example, despite there being more than double the number of patients afflicted with sickle cell disease (SCD) than patients with cystic fibrosis (CF), CF receives more than three times the funding from the National Institutes of Health than SCD, and hundreds of times more in private funding. However, SCD is more common in black patients, while CF is more common in white patients, which highlights a racial funding disparity for research within this space, which will inevitably also impact the development of AI for clinical workflow and diagnostics. The same funding patterns could also be observed between male- and female-associated disease burdens, typically offering more funding for research and development associated with the former (37).

Furthermore, measurement bias can occur when medical devices or methods used to gather data are themselves biased. Tools like pulse oximeters have been shown to overestimate oxygen levels in patients with darker skin, especially in women. Thus, datasets that use pulse oximeter data can be used to train many clinical support algorithms and feed inaccurate perspectives on basis of skin color and gender, further compromising how information is presented in clinical summaries (38).

Addressing bias in AI-driven patient chart review is crucial to ensuring equitable healthcare outcomes. The disparities in data representation, diagnostic practices, and resource allocation all contribute to biased predictions that can widen the healthcare gap between different demographic groups. As AI becomes more integrated into clinical decision-making, these biases can significantly affect how healthcare is delivered, particularly for underrepresented populations. To mitigate these issues, there is a need for more inclusive datasets, equal access to healthcare technology, and increased attention to funding disparities. Only through addressing these systemic biases can AI-driven tools become reliable allies in promoting health equity across diverse patient populations.

### 6.2 Challenges of cloud-based AI 1: patient-sided data privacy and informed consent

Currently, many general AI tools are hosted by cloud service providers (CSPs), partially due to the need for significant investments for proper local storage and computational capabilities. Cloud-based AI systems can process vast amounts of medical records, enhancing patient chart reviewing capabilities and aiding in better healthcare decision-making. However, this reliance on cloud platforms introduces significant risks, particularly concerning data privacy, informed consent, and cybersecurity.

One of the key issues with regulating the flow of information in AI is that attaching proper safeguards is difficult. It is fundamentally unknowable how data is processed and mutated for output generation. In fact, this is further complicated by the fact that most of the hardware and software that AI relies on is owned by ”big tech.” It further raises questions on data collection practices, and maintenance of privacy, and most importantly, informed consent. A key example is the controversy surrounding Alphabet’s Deepmind in 2017, where patient information was obtained without proper informed consent for the testing of their acute kidney injury algorithms (39, 40). In 2018, Deepmind came under fire again for migrating Streams, their clinical information management and decision support tool, to Google’s control without consent (41). These episodes exemplify the problems associated with privacy when utilizing data for machine learning and AI at a large scale, and highlight an important concept - informed consent.

Informed consent is a central ethical issue when using patient data for AI-driven clinical support. Traditional models of informed consent are being challenged by the scale and complexity of data used in AI. Patients often are unaware of how their data is used in secondary applications such as AI training, leading to potential ethical lapses. To address this, some researchers such as Wang et al. propose dynamic consent models in both the U.S. and China, where patients are regularly informed and can withdraw their consent as new AI applications emerge. This ensures patient agency in controlling how their personal health data is used. In countries like the United States and Europe, informed consent regulations such as HIPAA and GDPR are further being modified to manage the growing influence of AI data usage in healthcare (42).

### 6.3 Challenges of cloud-based AI 2: cybersecurity and adversarial attacks

However, even if patient-sided precautions can be established in an airtight manner, hosting cloud-based architecture still predisposes patient health information to greater cybersecurity risk. Cyberattacks for information theft are devastating but nothing new in the world of healthcare - from 2009 to 2023, over 500 million records have been subjected to data breaches, and various cyberattacks on covered entities have resulted in tens of millions of records stolen even in 2024 (43).

The deeper challenge that arises with the advent of LLMs is the threat of adversarial attacks, or the use of malicious prompt engineering to induce harmful outputs, including the disclosure of sensitive patient health information. Zhang et al. reveals an interesting example using a study on partially synthetic medical data. Partially synthetic data created by generative models is particularly vulnerable to adversarial attacks that reveal knowledge of whether a specific individual’s data contributed to the synthetic dataset. This creates a data breach risk, revealing private health information. The researchers used two datasets — Vanderbilt University Medical Center (VUMC) and the NIH-sponsored All of Us Research Program — to test these vulnerabilities, and these ”membership inference attacks” could reach up to 80% precision in identifying individuals from partially synthetic data. These breaches are especially harmful for individuals with pre-existing conditions. While these risks could be mitigated by techniques such as contrast representation learning, there is still a moral dilemma associated with the risks of using partially synthetic data vs. fully synthetic data, which has shown to be much more resistant to these types of attacks. The research stresses the importance of evaluating privacy risks before releasing synthetic health data and suggests that privacy protection methods, like differential privacy, could potentially reduce these risks, though they may also reduce data utility (44).

Several case studies have also presented another critical risk: the susceptibility of medical LLMs to data poisoning attacks. Data poisoning attacks occur when malicious inputs are introduced during the training phase, leading the model to produce skewed or incorrect results during clinical summarization or diagnosis tasks. This can have dangerous consequences in a healthcare setting, not only leading to harmful outputs, but also potentially disclosing sensitive patient data through manipulated queries. A key study by Abdali et. al demonstrated that injecting just a small number of poisoned examples into the LLM’s training data could cause the model to unintentionally leak patient details from its training set during inference (45).

But to present a contrasting view to this risk, another study by Yang et al. showed that adversarial attacks are not fully effective unless the adversarial data injection reaches a critical mass for domain-specific tasks such as prescribing medications. Thus, LLMs could be relatively safe from data poisoning attack motifs such as posting poisonous/malicious content online that many commercial open source LLMs are trained on (46).

These findings are a few but many of the growing field of data security associated with LLMs. They underscore the urgent need for robust defensive mechanisms in healthcare AI applications, such as fine-tuning models with adversarial training, using differential privacy techniques, and continuously auditing AI models for potential vulnerabilities. As medical AI continues to grow, securing LLMs against these types of attacks will be critical to protecting patient privacy and ensuring the safe deployment of AI in clinical environments.

## 7 Moving forward

Looking to the future, the integration of AI in patient chart review is poised to revolutionize healthcare workflows. Throughout this review, we have presented statistics in how patient chart reviewing has impacted both the workflow for clinicians and quality of care for patients through various mechanisms. AI capable of conducting patient chart review are undoubtedly poised to save invaluable amounts of time and energy for overworked clinicians, become employed in a plethora of different contexts and practices, and even boost quality of care, speed of healthcare delivery, and accuracy of disease management.

In 2024, integrating AI into clinical workflows presents a significant challenge, especially when considering the need for solutions that are compatible with over 600 EHR systems. A practical approach for clinicians may begin with adopting AI-powered scribes, followed by implementing automation tools for patient chart review. The initial integration may face considerable obstacles with considerable time being spent in proper management of patient data, troubleshooting the accuracy and utility of AI, and the time spent by clinicians in training and familiarization of new digital health tools. Making sure not to take shortcuts but properly handle each of those challenges through sufficient time and collaboration between AI engineers, healthcare professionals, and regulatory experts is paramount for the success of adoption. However, as more and more clinicians insert AI into their practice, growth can become exponential. In fact, additional EHR tasks, such as billing, lab orders, prescriptions, referrals, and patient communications, can also be handled by AI. With advancements in regulation-approved diagnostic AI, real-time management support may soon become a reality, accelerating innovation in healthcare.

However, it is crucial that ongoing efforts focus on addressing key limitations and barriers, particularly in terms of data privacy and security, as they are summarized in Table 2. Ensuring that AI models are trained on diverse, representative datasets will be vital in reducing bias and improving healthcare outcomes across different populations. Continued collaboration between healthcare providers, AI developers, and policymakers will help develop safeguards, ensuring that AI solutions promote equity and inclusivity.

**Table 2:** Barriers against effective AI-implementation in patient chart review.

Barriers against AI	Potential solutions
Comprehensiveness of information of patient summaries	Continued innovation in prompting to ensure information capture.
Accuracy of patient summary information without hallucinations	Continued innovation in LLM performance. Increased data availability from various sources to ensure both comprehensive capture of patient scenarios, decreased bias.
Implementation of AI-powered patient chart reviewing into the almost infinite, diverse needs of each clinician or practice	Training of fine-tuned children models to capture various specialties and practices. Tailored prompting.
Variability of AI outputs for similar patient scenarios	Continued innovation in LLM-output consistency using solutions such as soft-prompting.
Data availability: patient diversity, bias, or lack of data (especially in regards to US or China-centrism of data)	Increased data collection from sources typically underrepresented, and ensuring that data collection methods themselves are unbias (or at least accounted for). Increased focus on populations outside of U.S. and China.
Patient-sided privacy and informed consent for training new tools	Newer models of informed consent on data-side, such as dynamic consent models. Patient access and information to tools developed from their data.
Data extraction attacks from LLMs and cloud-based infrastructure security	Continued innovation in defense against typical cloud-based cybersecurity risks and adversarials attacks against LLMs.

Furthermore, as AI becomes more embedded in healthcare workflows, the importance of safeguarding patient privacy through secure cloud-based infrastructure and informed consent mechanisms cannot be overstated. Dynamic consent models and advanced encryption techniques must become standard practices to ensure that patient data is not only protected but also used ethically. Investments in securing cloud platforms, particularly with a focus on mitigating risks from adversarial attacks, will help ensure the sustainability and trustworthiness of AI-driven healthcare systems.

Moving forward, research into adversarial attacks and their potential to expose patient health data underscores the necessity for enhanced cybersecurity protocols in AI healthcare systems. As these models grow in complexity, healthcare institutions must prioritize building robust defenses, such as adversarial training and differential privacy, to mitigate the risks of data breaches. With the right regulatory oversight, secure cloud environments, and ongoing innovations, AI in clinical summarization can continue to develop, offering unprecedented improvements in patient care while maintaining ethical and privacy standards.
